# Association of Peroxisome Proliferator-Activated Receptor α/δ/γ With Obesity, and Gene–Gene Interaction, in the Chinese Han Population

**DOI:** 10.2188/jea.JE20120110

**Published:** 2013-05-05

**Authors:** Wenshu Luo, Zhirong Guo, Ming Wu, Chao Hao, Xiaoshu Hu, Zhengyuan Zhou, Zhiwu Zhou, Xingjuan Yao, Lijun Zhang, Jingchao Liu

**Affiliations:** 1 Changzhou Center for Disease Control and Prevention, Changzhou, Jiangsu, China; 2 Department of Epidemiology, School of Public Health, Soochow University, Suzhou, Jiangsu, China; 3 Center for Disease Control of Jiangsu Province, Nanjing, Jiangsu, China; 4 Health Bureau of Jiangsu Province, Nanjing, Jiangsu, China; 5 Center for Disease Control of Changshu, Suzhou, Jiangsu, China

**Keywords:** peroxisome proliferator-activated receptors, polymorphism, BMI, interaction

## Abstract

**Background:**

We investigated the association of 10 single-nucleotide polymorphisms (SNPs) in the peroxisome proliferator-activated receptors (PPARs) with obesity and the additional role of gene–gene interaction.

**Methods:**

Participants were recruited within the framework of the Prevention of Multiple Metabolic Disorders and MS in Jiangsu Province cohort population survey of an urban community in China. In total, 820 subjects (513 nonobese adults, 307 obese adults) were randomly selected, and no individuals were consanguineous. Ten SNPs (rs135539, rs4253778, rs1800206, rs2016520, rs9794, rs10865710, rs1805192, rs709158, rs3856806, and rs4684847) were genotyped and analyzed.

**Results:**

After covariate adjustment, minor alleles of rs2016520 in PPARδ and rs10865170 in PPARγ were associated with lower BMI (*P* < 0.01 for all). Generalized multifactor dimensionality reduction analysis showed significant gene–gene interaction among rs2016520, rs9794, and rs10865170 in 3-dimensional models (*P* = 0.0010); prediction accuracy was 0.6011 and cross-validation consistency was 9/10. It also showed significant gene–gene interaction between rs2016520 and rs10865170 in all 2-dimensional models (*P* = 0.0010); prediction accuracy was 0.6072 and cross-validation consistency was 9/10.

**Conclusions:**

rs2016520 and rs10865170 were associated with lower obesity risk. In addition, interaction was identified among rs2016520, rs9794, and rs10865170 in obesity.

## INTRODUCTION

Obesity is a complex metabolic disorder that affects a growing number of people worldwide^1^ and is the result of both genetic and environmental factors. Studies of twins^2^^,^^3^ indicate that genetic factors play a dominant role in determining body mass index (BMI), based on data from individuals in the same environmental setting.

The first genetic sensor for fats was identified in the early 1990s and was termed peroxisome proliferator-activated receptor α (PPARα) because of its ability to bind chemicals that induce peroxisome proliferation.^4^ Subsequent studies identified 2 additional, related receptors, known as PPARγ and PPARδ (also called PPARβ).^5^^,^^6^ As members of the nuclear receptor superfamily, PPARs act by controlling networks of target genes, and they have helped uncover the complex transcriptional underpinnings of metabolism.^7^ The 3 PPAR family members have distinct patterns of tissue distribution and, like typical siblings, are often functionally at odds with each other. Whereas PPARα and PPARγ are predominant in liver and adipose tissue, respectively, PPARα is involved in fat metabolism and fatty acid oxidation whereas PPARγ influences adipocyte differentiation and insulin action. PPARδ is abundantly expressed throughout the body but at low levels in liver; however, its function is not yet fully understood. Consistent with their expression profiles, the PPARs have unique functions in regulating energy metabolism. A number of polymorphisms^8^^–^^12^ have been described in the association of the PPARα and PPARγ isoforms with obesity. In contrast, few studies have focused on PPARδ, which is ubiquitously expressed, and the results have been inconsistent.^12^^–^^14^ Interaction between PPARδ and PPARα genotypes was demonstrated in a group of healthy men of normal weight.^14^ However, because obesity may result from the combined action of 2 or more single-nucleotide polymorphisms (SNPs), it is unclear whether corresponding gene–gene interactions exist among the 3 PPAR isoforms. Therefore, we studied a group of 820 adults to investigate the association of 10 SNPs of PPARα/δ/γ with BMI and the additional interaction among the 10 SNPs.

## METHODS

### Subjects

Participants were recruited within the framework of the PMMJS (Prevention of Multiple Metabolic Disorders and MS in Jiangsu Province) cohort population,^15^ which started from April 1999 to June 2004. In the present study, 4582 subjects with a follow-up time of 5 years received additional follow-up from March 2006 to October 2007. A total of 4083 participants (89.11%) completed the supplementary follow-up examination (those who attended the follow-up examination were similar to those who were lost to follow up in terms of age, sex, smoking status, alcohol consumption, family disease history, and metabolic variables, *P* > 0.05). After excluding subjects with a history of stroke/cardiovascular disease (*n* = 36) or diabetes mellitus (*n* = 289) and those with missing data (*n* = 133) or a BMI less than 18.5 kg/m^2^ (*n* = 27), simple random sampling was used to select 820 subjects (270 men, 550 women; 513 nonobese adults and 307 obese adults) from the remaining 3731 subjects. No 2 individuals were consanguineous. Those who were selected were similar to those who were not selected in terms of age, sex, smoking status, alcohol consumption, family disease history, and metabolic variables. A blood sample was collected at baseline from the 820 subjects and analyzed for genotype. The study outcome was overweight/obesity. BMI was recorded during follow up, and overweight/obesity was defined by using the World Health Organization (WHO) criteria for Asian populations, namely, a BMI of 24 kg/m^2^ or higher.^16^ This study was approved by the ethics committee of Soochow University.

### Anthropometric measurements

Data on demographics and lifestyle risk factors of all participants were obtained by using a standard questionnaire administered by trained staff. Body weight, height, and waist circumference were measured according to standardized procedures,^17^ and BMI was calculated as weight in kilograms divided by the square of the height in meters. Blood samples were collected in the morning after at least 8 hours of fasting. All plasma and serum samples were frozen at −80°C until laboratory testing. Fasting plasma glucose (FPG) was measured using an oxidase enzymatic method. Concentrations of high-density lipoprotein (HDL)-cholesterol and triglyceride (TG) were assessed enzymatically by an automatic biochemistry analyzer (Hitachi Inc, Tokyo, Japan) using commercial reagents. All laboratory analyses was performed at the same laboratory. The method of investigation during follow-up was the same as that used at baseline.

### SNP selection, genomic DNA extraction, and genotyping

We selected 10 SNPs within the PPARα/δ/γ gene based on (1) previously reported associations with metabolic abnormalities, (2) known heterozygosity and a minor allele frequency (MAF) greater than 0.05, and (3) whether the SNP was located in a gene fragment that could have functional effects.

Genomic DNA from participants was extracted from ethylenediaminetetraacetic acid (EDTA)-treated whole blood, using the DNA Blood Mini Kit (Qiagen, Hilden, Germany) according to the manufacturer’s instructions. Two approaches were used to analyze frequent and minor alleles for the 10 SNPs (rs135539, rs4253778, rs1800206, rs2016520, rs9794, rs10865710, rs1805192, rs709158, rs3856806, and rs4684847). Rs4253778 was detected by polymerase chain reaction-restriction fragment length polymorphisms (PCR-RFLP), and the 9 remaining SNPs were detected by TaqMan fluorescence probe. A restriction enzyme was used to identify and cut specific sequences, after which PCR was performed with the following primers: forward 5′-ACA ATC ACT CCT TAA ATA TGG TGG-3′ and reverse 5′-AAG TAG GGA CAG ACA GGA CCA GTA-3′. A 25-µl reaction mixture was amplified by PCR, including DNA 20 ng, 0.05 µl Ex Taq 1 DNA polymerase, 1 µl 10×buffer, 0.8 µl dTNP, 0.1 µl forward primers, and 0.1 µl reverse primers. PCR conditions were as follows: initial denaturation for 3 minutes at 95°C, denaturation for 10 seconds at 95°C, annealing for 30 seconds at 63°C, and extension for 30 seconds at 72°C, for 40 cycles. The ABI Prism 7000 software and an allelic discrimination procedure were used for genotyping the above-mentioned 9 SNPs (the probe sequences are shown in Table 1). The 25-µl reaction mixture included 1.25 µl SNP Genotyping Assays (20×), 12.5 µl Genotyping Master Mix (2×), and 20 ng DNA. The conditions were as follows: initial denaturation for 10 minutes at 95°C, denaturation for 15 seconds at 92°C, and annealing and extension for 90 seconds at 60°C, for 50 cycles.

**Table 1.** Description of the 10 SNPs and probe sequences of the 9 SNPs used in TaqMan fluorescence probe analysis

SNP ID	SNP	Chromosome	Position	Exon/Intron	Nucleotide substitution	Probe sequence
PPARα						
rs135539	1A>C	22	25949836	Intron_1	A>C	5′-AGCAGAATTTAAATCCTAGGTGATT[A/C] TTAACTCTAATCATACATCTAATGA-3′
rs4253778	7G>C	22	26021203	Intron_7	G>C	—
rs1800206	L162V	22	26004843	Exon_5	C>G	5′-CCAGTATTGTCGATTTCACAAGTGC[C/G] TTTCTGTCGGGATGTCACACAACGG-3′
PPARδ						
rs2016520	−87T>C	6	35318778	Exon-4	T>C	5′-CGGCCACATGCCGCGTCCCTGCCCC[C/G] ACCCGGGTCTGGTGCTGAGGATACA-3′
rs9794	2806C>G	6	35335795	Exon-9	C>G	5′-CCTCTGCCCAGGCTGATGGGAACCA[C/T] CCTGTAGAGGTCCATCTGCGTTCAG-3′
PPARγ						
rs709158	Intron A>G	3	12403176	Intron_2	A>G	5′-AGATACGGGGGAGGAAATTCACTGG[A/G] TTTTACAATATATTTTTCAAGGCAA-3′
rs10865710	C681G	3	12293198	Exon_A2	C>G	5′-TTGGCATTAGATGCTGTTTTGTCTT[C/G] ATGGAAAATACAGCTATTCTAGGAT-3′
rs1805192	Pro12Ala	3	12361238	Exon_B	C>G	5′-ACCTCAGACAGATTGTCACGGAACA[C/T] GTGCAGCTACTGCAGGTGATCAAGA-3′
rs4684847	Intron C>T	3	12326337	Intron_3	C>G	5′-ATTTATTTAAATCATCTCTAATTCT[C/T] ACAACTCCGAAAAGATAAGAAAACA-3′
rs3856806	C161T	3	12415557	Exon-6	C>T	5′-GGTTGACACAGAGATGCCATTCTGG[C/G] CCACCAACTTTGGGATCAGCTCCGT-3′

SNP, single-nucleotide polymorphism.

### Statistical analysis

The mean and SD for normally distributed continuous variables, and percentages for categorical variable, were calculated and compared between obese and nonobese participants. Median and interquartile range were calculated for continuous variables that were not normally distributed. Differences in the characteristics of obese and nonobese participants were examined by using 1-way ANOVA, the rank test, and the χ^2^ test. For the purpose of quality control, deviation from the Hardy-Weinberg equilibrium (HWE) was used to detect genotype typing errors by Fisher's exact test, using the program HWE.^18^^,^^19^ Linkage disequilibrium (LD) between polymorphisms was estimated by using SHEsis (available online at http://analysis.bio-x.cn). A logistic regression model was used to examine the association between PPAR polymorphisms and obesity, and odds ratios (ORs) and 95% CIs were calculated. Odds were adjusted for potential confounding factors such as sex, age, smoking and alcohol consumption status, high-fat diet, low-fiber diet, occupational activity, FPG, TG, and HDL-C.

Generalized multifactor dimensionality reduction (GMDR)^20^ analysis was used to analyze interaction among the 10 SNPs. To assess each selected interaction, parameters were calculated, including cross-validation consistency, testing-balanced accuracy, and the sign test. The cross-validation consistency score is a measure of the degree of consistency with which the selected interaction is identified as the best model among all possibilities considered. Testing-balanced accuracy is a measure of the degree to which the interaction accurately predicts case–control status, and yields a score between 0.50 (indicating that the model predicts no better than chance) and 1.00 (indicating perfect prediction). Finally, the sign test, or permutation test (providing empirical *P*-values), for prediction accuracy can be used to measure the significance of an identified model. In this study, we analyzed interaction among the 10 SNPs by using a GMDR model that adjusted for sex, age, smoking status, alcohol status, high-fat diet, low-fiber diet, occupational activity, FPG, TG, and HDL-C.

## RESULTS

A total of 820 participants (270 men, 550 women) were studied, including 513 nonobese and 307 obese adults. Participant characteristics stratified by BMI are shown in Table 2. Mean HDL was significantly higher in obese subjects than in nonobese subjects (*P* = 0.01). Mean TG and FPG were also significantly higher in obese subjects than in nonobese subjects (*P* < 0.05 for both comparisons). The distributions of occupational activity, current smoking, and education status did not significantly differ between men and women (*P* > 0.05 for all comparisons).

**Table 2.** General characteristics of the 820 participants according to obesity status

Variables	Total (*n* = 820)	Nonobese (*n* = 513)	Obese (*n* = 307)	*P*-values
Males, *n* (%)	270 (32.9)	196 (32.8)	74 (33.2)	0.92
Age (years)	50.05 ± 9.41	50.49 ± 9.56	48.85 ± 8.92	0.03
Education status *n* (%)				0.70
Illiterate	287 (35.0)	214 (74.6)	73 (25.4)	
Elementary school	255 (31.1)	184 (72.2)	71 (27.8)	
Middle school or higher	278 (33.9)	199 (71.6)	79 (28.4)	
Income per month *n* (%)				0.80
<6000 RMB (¥)	564 (68.8)	411 (72.9)	153 (27.1)	
6000–<15 000 RMB (¥)	213 (26.0)	153 (71.8)	60 (28.2)	
≥15 000 RMB (¥)	43 (5.2)	33 (76.7)	10 (23.3)	
Current smoking *n* (%)	199 (24.3)	152 (25.5)	47 (21.1)	0.26
Current alcohol consumption *n* (%)	205 (25.0)	145 (24.3)	60 (26.9)	0.44
High-fat diet *n* (%)	235 (28.7)	171 (28.6)	64 (28.7)	0.99
Low-fiber diet *n* (%)	59 (7.2)	45 (7.5)	14 (6.3)	0.54
Occupational activity *n* (%)				0.21
100% mental work	53 (6.5)	42 (79.2)	11 (20.8)	
Mostly mental work	100 (12.2)	66 (66.0)	34 (34.0)	
Mostly physical work	407 (49.6)	304 (74.7)	103 (25.3)	
100% physical work	260 (31.7)	185 (71.2)	75 (28.8)	
FPG (mmol/L)	5.01 ± 0.75	4.98 ± 1.24	5.22 ± 1.26	0.02
TG (mmol/L)	1.27 (1.01–1.62)	1.26 (1.00–1.60)	1.32 (1.05–1.67)	<0.001
TC (mmol/L)	4.90 ± 1.12	4.87 ± 1.12	5.00 ± 1.10	0.16
HDL-C (mmol/L)	1.29 ± 0.30	1.30 ± 0.30	1.24 ± 0.28	0.01

Values are median and interquartile range for TG and means ± SD for age, FPG, TC, HDL-C;

TC, total cholesterol; HDL, high-density lipoprotein; LDL, low-density lipoprotein; FPG, fasting plasma glucose; TG, triglyceride.

All genotypes were distributed according to the Hardy–Weinberg equilibrium. There were significant differences in the rs2016520 and rs10865710 alleles and genotype distributions between obese and nonobese participants (Table 3). The frequency of the C allele of rs2016520 was higher in nonobese participants (26.2% in obese subjects vs 32.8% in nonobese subjects, *P* = 0.001). In contrast, the frequency of the C allele of rs10865710 was lower in non-obese adults (36.8% in obese subjects vs 30.7% in nonobese subjects, *P* = 0.01). Odds ratios showed an association of genotypes of variants in rs2016520 and rs10865710 with decreased obesity risk, after adjustment for all confounders: obesity risk was significantly higher in individuals with rs2016520-CC, rs2016520-CT, and rs10865710-GG homozygotes (*P* < 0.05 for all comparisons). Carriers of the C allele of the rs2016520 polymorphism had a lower obesity risk than did those with the TT variant (CC+CT vs TT; adjusted OR = 0.63, 95% CI = 0.48–0.87; *P* = 0.003). However, the other 8 SNPs in PPARs were not significantly associated with obesity before or after covariate adjustment.

**Table 3.** Genotype and allele frequencies for the 10 SNPs in the PPAR gene according to obesity status

SNPs	Genotypes and alleles	Frequencies *n* (%)		OR (95% CI)^a^	*P*-values

		Nonobese subjects (*n* = 513)	Obese subjects (*n* = 307)		
PPARα					
rs135539	AA	294 (57.3)	190 (61.9)	1.00	—
	AC	188 (36.6)	91 (29.6)	0.94 (0.55–1.62)	0.82
	CC	31 (6.1)	26 (8.5)	0.72 (0.41–1.28)	0.27
	AC+CC	219 (42.7)	117 (38.1)	0.80 (0.60–1.08)	0.15
	A	776 (75.5)	471 (76.7)		0.47
	C	252 (24.5)	143 (23.3)		
rs4253778	GG	392 (76.4)	223 (72.6)	1.00	—
	GC	114 (22.2)	69 (22.5)	1.12 (0.79–1.59)	0.52
	CC	7 (1.4)	15 (4.9)	1.75 (0.84–3.65)	0.30
	GC+CC	121 (23.6)	84 (27.4)	1.18 (0.85–1.65)	0.31
	G	898 (87.5)	515 (83.9)		0.12
	C	128 (12.5)	99 (16.1)		
rs1800206	LL	386 (75.2)	236 (76.9)	1.00	—
	LV	123 (24.0)	68 (22.1)	0.91 (0.64–1.31)	0.61
	VV	4 (0.8)	3 (1.0)	1.30 (0.28–5.89)	0.74
	LV+VV	127 (24.8)	71 (23.1)	0.90 (0.63–1.30)	0.59
	L	895 (87.2)	540 (87.9)		0.67
	V	131 (12.8)	74 (11.1)		
PPARδ					
rs9794	CC	304 (59.3)	194 (63.2)	1.00	—
	CG	184 (35.9)	98 (31.9)	0.82 (0.60–1.12)	0.21
	GG	25 (4.9)	15 (4.9)	0.90 (0.46–1.75)	0.74
	CG+GG	209 (40.8)	113 (36.8)	0.83 (0.62–1.13)	0.22
	C	792 (77.2)	486 (79.2)		0.35
	G	234 (22.8)	128 (20.8)		
rs2016520	TT	223 (43.5)	165 (53.7)	1.00	—
	TC	243 (47.4)	123 (40.1)	0.67 (0.49–0.92)	0.01*
	CC	47 (9.2)	19 (6.2)	0.54 (0.30–0.95)	0.03*
	TC+CC	290 (56.6)	142 (46.3)	0.63 (0.48–0.87)	0.003*
	T	689 (67.2)	453 (73.8)		0.001*
	C	337 (32.8)	161 (26.2)		
PPARγ					
rs10865710	CC	240 (46.8)	127 (41.4)	1.00	—
	CG	231 (45.0)	134 (43.6)	1.07 (0.78–1.46)	0.67
	GG	42 (8.2)	46 (15.0)	2.18 (1.34–3.55)	0.002*
	CG+GG	273 (53.2)	180 (58.6)	1.23 (0.92–1.65)	0.17
	C	711 (69.3)	388 (63.2)		0.01*
	G	315 (30.7)	226 (36.8)		
rs3856806	CC	259 (50.5)	159 (51.8)	1.00	—
	CT	208 (40.5)	118 (38.4)	0.89 (0.64–1.28)	0.54
	TT	46 (9.0)	30 (9.8)	1.03 (0.59–1.73)	0.92
	CT+TT	254 (49.5)	148 (48.2)	0.92 (0.69–1.26)	0.66
	C	726 (70.8)	436 (71.0)		0.91
	T	300 (29.2)	178 (29.0)		
rs709158	AA	261 (50.9)	149 (48.5)	1.00	—
	AG	209 (40.7)	125 (40.7)	1.01 (0.74–1.37)	0.95
	GG	43 (8.4)	33 (10.7)	1.31 (0.79–2.17)	0.30
	AG+GG	252 (49.1)	158 (51.4)	1.06 (0.79–1.42)	0.69
	A	731 (71.2)	423 (68.9)		0.31
	G	295 (28.8)	191 (31.1)		
rs1805192	PP	283 (55.2)	176 (57.3)	1.00	—
	PA	194 (37.8)	101 (32.9)	0.88 (0.64–1.21)	0.44
	AA	36 (7.0)	30 (9.8)	1.14 (0.65–1.96)	0.63
	PA+AA	230 (44.8)	131 (42.7)	0.93 (0.69–1.25)	0.62
	Pro	760 (74.1)	453 (73.8)		0.86
	Ala	266 (25.9)	161 (26.2)		
rs4684847	CC	324 (63.2)	195 (63.5)	1.00	—
	CT	162 (31.6)	95 (30.9)	1.00 (0.73–1.37)	0.98
	TT	27 (5.3)	17 (5.5)	1.01 (0.53–1.92)	0.99
	CT+TT	189 (36.9)	112 (36.4)	1.00 (0.73–1.35)	0.98
	C	810 (78.9)	485 (79.0)		0.98
	T	216 (21.1)	129 (21.0)		

^a^Adjusted for sex, age, smoking and alcohol status, high-fat diet, low-fiber diet, occupational activity, FPG, TG, and HDL-C.

*P-values less than 0.05 were considered statistically significant.

Pairwise LD analysis between SNPs was measured, and D′ was less than 0.75 in all cases. We then used GMDR analysis to assess the effect of interaction among the 10 SNPs, after adjustment for all covariates. Table 4 summarizes the results obtained from GMDR analysis for 2- to 9-locus models after covariate adjustment. There was a significant (*P* = 0.0010) 2-locus model involving rs2016520 and rs10865170, indicating potential gene–gene interaction between rs2016520 and rs10865170. There was a significant (*P* = 0.0010) 3-locus model involving rs2016520, rs9794, and rs10865170, indicating potential gene–gene interaction among rs2016520, rs9794, and rs10865170. Overall, the 2- and 3-locus models had a cross-validation consistency of 9 of 10 (for both) and testing accuracies of 60.72% and 60.11%, respectively.

**Table 4.** Best gene–gene interaction models, as identified by GMDR

Locus no.	Best combination	Cross-validation consistency	Testing accuracy	*P*-values^a^
2	rs2016520 rs10865170	9/10	0.6072	0.0010
3	rs2016520 rs9794 rs10865170	9/10	0.6011	0.0010
4	rs2016520 rs9794 rs10865170 rs1805192	6/10	0.5399	0.0547
5	rs9794 rs10865170 rs3856806 rs1805192 rs4684847	5/10	0.4958	0.1719
6	rs135539 rs9794 rs10865170 rs3856806 rs1805192 rs4684847	3/10	0.4958	0.4258
7	rs135539 rs2016520 rs10865170 rs3856806 rs709158 rs1805192 rs4684847	5/10	0.4958	0.6230
8	rs135539 rs9794 rs2016520 rs10865170 rs3856806 rs709158 rs1805192 rs4684847	6/10	0.4958	0.9893
9	rs135539 rs4253778 rs9794 rs2016520 rs10865170 rs3856806 rs709158 rs1805192 rs4684847	8/10	0.5399	0.3770

^a^Adjusted for sex, age, smoking and alcohol status, high-fat diet, low-fiber diet, occupational activity, FPG, TG, and HDL-C.

To obtain ORS and 95% CIs for the joint effects of candidate SNPs (rs2016520 and rs10865170; rs2016520, rs9794, and rs10865170) on obesity, we conducted interaction analysis among SNPs in the 2- and 3-locus models. Table 5 summarizes the results obtained from interaction analysis of the 2- and 3-locus models after covariate adjustment. In the 2-locus model, subjects with the rs2016520-TC or CC and rs10865170-CG or GG genotypes had the lowest obesity risk (RR, 0.42; 95% CI, 0.26–0.70; *P* < 0.01) as compared with subjects with the rs2016520-TT and rs10865170-CC genotypes. In the 3-locus model, subjects with the rs2016520-TC or CC, rs10865170-CC, and rs9794-CG or GG genotypes had lowest obesity risk (RR, 0.25; 95% CI, 0.12–0.57; *P* < 0.001) as compared with subjects with the rs2016520-TT, rs10865170-CG or GG, and rs9794-CC genotypes.

**Table 5.** Interaction analysis for 2- and 3-locus models, by logistic regression

rs2016520	rs10865710	rs9794	RR (95% CI)^a^	*P*-values
2-locus model				
TT	CC		1.00	—
TC or CC	CC		0.46 (0.30–0.72)	<0.001
TT	CG or GG		0.67 (0.41–1.09)	0.09
TC or CC	CG or GG		0.42 (0.26–0.70)	<0.01
3-locus model				
TT	CG or GG	CC	1.00	—
TC or CC	CC	CC	0.50 (0.28–0.94)	0.03
TT	CC	CG or GG	0.44 (0.21–0.95)	0.03
TC or CC	CG or GG	CG or GG	0.61 (0.30–1.24)	0.19
TT	CC	CC	1.13 (0.64–2.02)	0.71
TC or CC	CG or GG	CC	0.45 (0.24–0.86)	0.01
TT	CG or GG	CG or GG	0.92 (0.51–1.69)	0.87
TC or CC	CC	CG or GG	0.25 (0.12–0.57)	<0.001

^a^Adjusted for sex, age, smoking and alcohol status, high-fat diet, low-fiber diet, occupational activity, FPG, TG, and HDL-C.

## DISCUSSION

The results of this study showed that the rs2016520 minor allele (C allele) of PPARδ was significantly associated with lower BMI. The frequency of the C allele was 30.4% in the present population, which is similar to the proportion in the Han population (30.7%) of Dalian reported by Yu,^21^ higher than that in Korean^13^ and Swedish populations,^22^ and lower than that in Scotland.^23^ Previous studies indicated that PPARδ was involved in adipocyte differentiation and insulin action. In an animal model, Wang et al^24^ suggested that activation of PPARδ through a selective agonist reduced fatty acid storage in adipocytes and prevented development of obesity in animals fed a high-fat diet. Oliver et al^25^ suggested that treatment of obese rhesus monkeys with the selective PPARδ agonist GW501516 significantly improved metabolic traits by increasing HDL and lowering LDL, TG, and insulin. Aberle et al^14^ found that the C allele of PPARδ was significantly associated with lower BMI. Our results are similar to those of the above-mentioned studies.

Previous studies suggest that PPARγ is a strong candidate gene for predisposition to obesity, via increased adiposity.^26^^,^^27^ PPARγ is expressed almost exclusively in adipose tissue and determines adiposity by regulating adipocyte differentiation and fat metabolism through a complex program of gene expression. PPARγ appears therefore to be a key regulator of adiposity and energy balance and may be one of the most important genetic factors in predisposing individuals to obesity. A recent study showed that PPARγ knockout mice fail to develop adipose tissue, which demonstrates that the PPARγ gene is essential in forming new adipocytes.^28^ In humans, PPARγ mRNA levels are higher in adipocytes from morbidly obese subjects,^29^ whereas PPARγ expression is attenuated in visceral adipose tissue from lean subjects.^30^ PPARγ therefore represents a direct genetic link to regulation of regional adiposity and body weight. More recently, a variant (rs10865170) that resides in the PPARγ3 promoter region has been described.^31^ This variant has been implicated in modulating bone growth^32^ via direct influence on growth factor signaling in bone and child height.^33^ In our study, we noted a significant association only between rs10865170 in PPARγ and obesity.

A recent report suggested that genetic susceptibility to obesity was related to multiple genes, most of which were minor genes. Because of the distance among genes, epistasis^34^ exists among PPARs genotypes and other obesity-related genes. For this reason, an interaction analysis of 10 SNPs was needed. We used GMDR analysis to assess interaction among the 10 SNPs on obesity risk after covariate adjustment. The results showed potential gene–gene interaction between rs2016520 and rs10865170 and among rs2016520, rs9794, and rs10865170. The 3-locus model was the best GMDR model. Previous evidence suggests that PPARγ rs10865170 and rs1805192 display opposing interaction in terms of growth phenotype^33^; however, in this study, no significant interaction was seen between rs10865170 and rs1805192. Studies^35^^,^^36^ showed that functional cross-talk between PPARs might exist in relation to control of their expression levels. In addition, some interplay between PPAR isoforms is suggested both by the repression of PPARγ- and PPARα-mediated activation of target gene expression after PPARδ activation and by PPARδ-dependent PPARγ activation. Wang et al^24^^,^^37^ indicated that PPARδ stimulates expression of PPARγ coactivator 21α (PGC21 α), which is highly valuable to consumption of energy in organisms and suppression of fat accumulation. In addition, rs9794 was not associated with BMI, but can significantly affect obesity risk when accompanied with rs2016520 and rs10865170. These findings indicate that a minor gene (even when its main effects are close to nil) can have a strong effect on obesity, due to the presence of gene–gene interaction.

The limitations of this study should be considered. First, only 1 to 5 SNPs per candidate gene were chosen. The selected SNPs were not sufficient to capture most of the genetic information of the candidate gene. Future studies should include more SNPs. Moreover, the functional relevance of our findings must be explored. Second, the present sample size was small, although the number of study participants met the requirement for analysis. Additional, larger sample studies should be conducted in the future.

In conclusion, our results show important associations of PPARδ rs2016520 and PPARγ rs10865170 with BMI, and the observed PPAR interactions have a combined effect on obesity due to gene–gene interaction among rs2016520, rs9794, and rs10865170.

## ONLINE ONLY MATERIALS

eTables. Association between 10 SNPs and obesity.
