# Supplementary material for: Association of Peroxisome Proliferator-Activated Receptor α/δ/γ With Obesity, and Gene–Gene Interaction, in the Chinese Han Population
Source: J Epidemiol. 2013 May 5;23(3):187–94. doi: 10.2188/jea.JE20120110 (PMC3700259; doi:10.2188/jea.JE20120110)
Supplement: eTables. — Association between 10 SNPs and obesity. [file je-23-187-s001.pdf]

eTable Association Between 10 SNPs and Obesity

SNP	Obesity					
	OR(95%CI)[1]	OR(95%CI)[2]	OR(95%CI)[3]	OR(95%CI)[4]	OR(95%CI)[5]	OR(95%CI)[6]
rs135539	0.827(0.619-1.104), P=0.197	0.818(0.612-1.093), P=0.175	0.804(0.600-1.077), P=1.143	0.815(0.607-1.093), P=0.172	0.811(0.605-1.089), P=0.163	0.802(0.596-1.078), P=0.143
rs4253778	1.220(0.883-1.686), P=0.227	1.220(0.882-1.687), P=0.229	1.214(0.876-1.683), P=0.243	1.216(0.877-1.686), P=0.240	1.210(0.873-1.679), P=0.253	1.189(0.854-1.654), P=0.306
rs1800206	0.914(0.656-1.275), P=0.598	0.913(0.654-1.274), P=0.591	0.899(0.642-1.259), P=0.535	0.948(0.666-1.349), P=0.765	0.919(0.643-1.341), P=0.643	0.909(0.631-1.309), P=0.606
rs9794	0.847(0.633-1.134), P=0.265	0.847(0.633-1.135), P=0.266	0.830(0.619-1.115), P=0.216	0.836(0.622-1.122), P=0.232	0.834(0.621-1.120), P=0.227	0.826(0.614-1.112), P=0.209
rs2016520	0.662(0.498-0.879), P=0.004	0.662(0.498-0.880), P=0.005	0.638(0.478-0.851), P=0.002	0.637(0.477-0.851), P=0.002	0.641(0.480-0.856), P=0.003	0.641(0.479-0.857), P=0.003
rs10865710	1.246(0.936-1.658), P=0.131	1.267(0.950-1.690), P=0.108	1.244(0.930-1.662), P=0.141	1.243(0.930-1.663), P=0.142	1.250(0.935-1.673), P=1.132	1.224(0.912-1.641), P=0.178
rs3856806	0.949(0.715-1.260), P=0.718	0.920(0.691-1.224), P=0.567	0.932(0.699-1.242), P=0.630	0.947(0.709-1.264), P=0.711	0.943(0.706-1.259), P=0.690	0.928(0.694-1.242), P=0.617
rs709158	1.098(0.828-1.457), P=0.516	1.093(0.822-1.453), P=0.542	1.051(0.788-1.402), P=0.734	1.053(0.789-1.406), P=0.724	1.048(0.785-1.399), P=0.751	1.061(0.793-1.419), P=0.692
rs1805192	0.916(0.688-1.218), P=0.546	0.893(0.670-1.190), P=0.440	0.904(0.676-1.208), P=0.494	0.929(0.690-1.251), P=0.628	0.926(0.688-1.247), P=0.926	0.926(0.686-1.249), P=0.616
rs4684847	0.985(0.734-1.321) , P=0.918	0.972(0.724-1.305), P=0.851	0.993(0.738-1.336), P=0.961	1.002(0.744-1.349), P=0.991	0.995(0.738-1.340), P=0.972	0.997(0.738-1.346), P=0.983

[1]Unadjusted

[2]Adjusted for sex and age

[3]Adjusted for sex, age, and smoking and alcohol status

[4]Adjusted for sex, age, smoking and alcohol status, high-fat diet, and low-fiber diet

[5]Adjusted for sex, age, smoking and alcohol status, high-fat diet, low-fiber diet, and occupational activity

[6]Adjusted for sex, age, smoking and alcohol status, high-fat diet, low-fiber diet, occupational activity, FPG, TG, and HDL-C
